# From global recommendations to (in)action: A scoping review of the coverage of companion of choice for women during labour and birth

**DOI:** 10.1371/journal.pgph.0001476

**Published:** 2023-02-01

**Authors:** Meghan A. Bohren, Alya Hazfiarini, Martha Vazquez Corona, Mercedes Colomar, Bremen De Mucio, Özge Tunçalp, Anayda Portela

**Affiliations:** 1 Gender and Women’s Health Unit, Centre for Health Equity, School of Population and Global Health, University of Melbourne, Carlton, Victoria, Australia; 2 The Latin American Center for Perinatology/Women´s and Reproductive Health Unit, Pan American Health Organization, Montevideo, Uruguay; 3 UNDP/UNFPA/UNICEF/WHO/World Bank Special Programme of Research, Development and Research Training in Human Reproduction (HRP), Department of Sexual and Reproductive Health and Research, World Health Organization, Geneva, Switzerland; 4 Department of Maternal, Newborn, Child and Adolescent Health and Ageing, World Health Organization, Geneva, Switzerland; American University of Beirut, LEBANON

## Abstract

Women greatly value and benefit from the presence of someone they trust to support them throughout labour and childbirth (‘labour companion of choice’). Labour companionship improves maternal and perinatal outcomes, including enhancing physiological labour and birth experiences. Despite clear benefits, implementation is slow. We conducted a scoping review to assess coverage and models of labour companionship, including quantitative studies reporting coverage of labour companionship in any level health facility globally. We searched MEDLINE, CINAHL, and Global Health from 1 January 2010–14 December 2021. We extracted data on study design, labour companionship coverage, timing and type of companions allowed, and recoded data into categories for comparison across studies. We included data from a maternal health sentinel network of hospitals in Latin America, using descriptive statistics to assess coverage among 120,581 women giving birth in these sites from April 2018-April 2022. In the scoping review, we included 77 studies from 27 countries. There was wide variation in the coverage of labour companionship: almost one-third of studies reported coverage less than 40%, and one-third of studies reported coverage between 40–80%. Husbands or partners were the most frequent companion (37.7%, 29/77), followed by family member or friend (gender not specified) (32.5%, 25/77), family member or friend (female-only) (13.0%, 10/77). Across nine sentinel hospitals in five Latin American countries, there was variation in coverage, with no companion at any time ranging from 14.9%-93.8%. Despite the well-known benefits and factors affecting implementation of labour companionship, more work is needed to improve equitable coverage. Concerted efforts are needed to engage with communities, health workers, health managers, and policy-makers to establish policies, address implementation barriers, and integrate data on coverage into perinatal records and quality processes to ensure that all women have access. Harmonized reporting of labour companionship would greatly enhance understanding at global level.

## Background

Research has consistently shown that women greatly value and benefit from the presence of someone they trust to support them throughout labour and childbirth [1]. Women who have a companion of choice during labour and birth (hereafter referred to as labour companion) report that this support helps them feel safe, strong, confident, and secure [2]. Some women may prefer their husband or partner as a companion and view this as a family bonding experience; other women may prefer a female relative or friend. There is evidence that labour companionship improves maternal and perinatal outcomes, including enhancing the physiological process of labour [1]. Research has shown clinically meaningful benefits of the support, including shorter duration of labour, increased rates of spontaneous vaginal birth, decreased caesarean section and intrapartum analgesia use, and increased satisfaction with childbirth experiences [1]. Women have also reported less fear and distress during labour, and babies are less likely to have low five-minute Apgar scores [1]. Based on this evidence, a companion of choice during labour and childbirth is recommended in three World Health Organization (WHO) guideline recommendations [3–5]. Moreover, the WHO *Standards for improving quality of maternal and newborn care in health facilities* includes a quality statement that every woman should be offered the option to experience labour and childbirth with the companion of her choice [6], and it is considered an important intervention to improve women’s experience of care.

Historically, global maternal health efforts have focused on addressing high rates of mortality, morbidity, and access barriers, particularly around improving access to facility-based maternal and newborn care services. However, the last decade has seen a shift in global maternal health efforts towards improving quality of maternity care services with a new focus on improving women’s and families’ experiences of care, equity, respect, and dignity. Despite clear evidence of benefit, no harms, and labour companionship being a critical component of quality and respectful maternity care, many national and facility-level policies do not allow for labour companionship. For example, the ‘WHO reproductive, maternal, newborn, child and adolescent health policy survey’ (2018) showed that while labour companionship was recommended in national policies and guidelines in most countries in the Americas, European, and Southeast Asian regions, approximately one-third of countries in the African region, and three-quarters of countries in the Eastern Mediterranean region had no national level policies or guidelines recommending labour companionship [7, 8].

Where policies do exist, labour companionship may be limited in implementation, due in part to health manager and health worker resistance to implementation [2]. More work is urgently needed to improve understanding of the global landscape of labour companionship as a priority woman-centred maternal health intervention, as well as implementation evidence about how to best design labour companionship models that account for context, and the values and preferences of key stakeholders, including women. This scoping review aims to identify data on coverage and describe the different models of labour companionship for women giving birth in health facilities globally.

## Methods

This study is reported per the Preferred Reporting Items for Systematic Reviews and Meta-Analyses extension for scoping reviews (PRISMA-ScR) [9] (S1 Appendix), and is registered with Open Science Foundation (Protocol ID: k69sg).

### Topic of interest and types of studies

The population of interest was women and birthing people with any mode of birth (vaginal birth, caesarean birth, assisted vaginal birth) in any type or level of health facility in any country globally. There were no restrictions on age, ethnicity, parity, obstetric history, or gestational age of the pregnancy.

Publications were eligible for inclusion if they provided data on coverage of labour companion (whether the objective was to measure coverage or the study had other objectives) and used quantitative methods for data collection and analysis or reporting (e.g., cross sectional survey with women during the postpartum period, observations of labour and birth). Publications were eligible for inclusion if they were published in any language, and from 2010 onwards in order to ensure findings reflect contemporary maternity care practices. Only publications with access to full text were included to ensure that we were able to extract important contextual data (e.g., excluding studies with only a conference abstract available).

### Search methods for identification of studies

We adapted the search strategy used for the 2019 Cochrane Qualitative Evidence Synthesis about labour companionship [2] for this review, and searched MEDLINE, CINAHL, and Global Health databases (S2 Appendix). The adaptations of this search strategy were to 1) remove qualitative research methodology filters, and 2) add date limiters to 1 January 2010 to the date of search (14 December 2021). The search terms related to “childbirth”, “social support”, and “labour companions”, with no limitations on language or geography.

### Selection of studies

We imported the search results into Covidence (Covidence systematic review software, Veritas Health Innovation, Melbourne, Australia), and at least two reviewers (MAB, AH, MVC) independently reviewed title and abstracts to evaluate eligibility against the prespecified criteria. Google Translate was used to translate titles and abstracts published in languages other than those the review team are proficient in (Bahasa Indonesia, English, French, Portuguese, Spanish, and Turkish). We retrieved the full text of all papers identified as potentially relevant and two reviewers assessed eligibility independently (AH, MVC), with disagreements resolved through discussion with a third reviewer (MAB, AP). If the translated title and abstracts were potentially relevant for inclusion, the full text was translated first using Google Translate, and then translation was checked and corrected by a native speaker (used for studies published in Mandarin and Persian).

### Data extraction and synthesis

Two reviewers (AH, MVC) extracted relevant data using a form designed and tested for this review, including information on the following domains of interest: study setting, study design, sample size and characteristics, data collection and analysis methods, type of health facility, coverage of labour companionship, type of labour companions allowed (by the health facility or health worker, such as labour companion of the woman’s choice, only female companions, only partners/husbands), timing labour companions are allowed by the health facility or health worker (e.g. labour, birth, postnatal, during caesarean birth). Where there was more than one time point of data (e.g., baseline and endline), we extracted baseline data only, and where there was in interventional component to the study with a control and intervention group, we extracted the control group data only, as the baseline and control data better reflect true coverage. Disagreements were resolved through discussion with a third reviewer (MAB, AP). Aligned with scoping review methods, we aimed to produce an overview of the coverage related to labour companionship, and therefore did not conduct critical appraisal.

During the charting stage of the synthesis, we extracted and recoded the data into meaningful categories for comparison across studies. We recoded the country setting into WHO regions (African, Americas, Eastern Mediterranean, European, Southeast Asian, Western Pacific), by country-income level at the time of publication (per World Bank classifications of high, upper-middle, lower-middle, low), and by urban/rural location. We recoded the type of labour companion allowed (by the health facility or health worker) to determine whether a companion of the woman’s choice was allowed (yes, no, not specified), and into comparable categories (any family member or friend, female family member or friend, husband or partner, doula, other, not specified). We recoded type of health facility as public, mixed public and non-public, non-public hospital, not applicable (population-based studies), and not specified. We recoded the coverage of labour companionship into quintiles for comparison (0–19.9%, 20.0–39.9%, 40.0–59.9%, 60.0–79.9%, 80.0–100%).

### Labour companionship coverage in a network of sentinel sites in Latin America

In addition to database searching, we contacted WHO regional advisors for maternal health to enquire about available coverage data related to labour companionship in each WHO region. From these discussions, we identified one multi-country sentinel dataset in Latin America. The Latin American Center for Perinatology / Women’s and Reproductive Health Unit (CLP/WR) is a regional hub for perinatal research within the Pan American Health Organization (PAHO) and the WHO office for the Americas Region. CLP runs a multi-country, multi-site maternal health sentinel network across nine health facilities in five Latin American countries (Bolivia, Dominican Republic, Guatemala, Honduras, and Nicaragua) since 2014. All the sites are public institutions with more than 2500 births a year. There are four second- and five third- level maternity hospitals and they all use a common data collection system called the Perinatal Information System (*Sistema Informático Perinatal* (SIP). SIP is a free perinatal clinical record system which includes a recording form for information from pregnancy to the postnatal period, and a platform where data can be entered (more information on SIP is available in detail elsewhere) [10]. The SIP clinical record includes a question on whether women were supported by a companion during labour only, during birth only, or during labour and birth, and who this person was: partner/husband, family, or other. Using data from April 2018 to April 2022, we descriptively analysed SIP data about the coverage of labour companion from a sample of 120,581 women from nine hospitals in five countries, which represents all women who gave birth in sentinel sites during this period. This analysis complements the systematic scoping review with robust observational sentinel data about the coverage of labour companionship in the Latin America region.

## Results

### Scoping review findings

**Fig 1:**
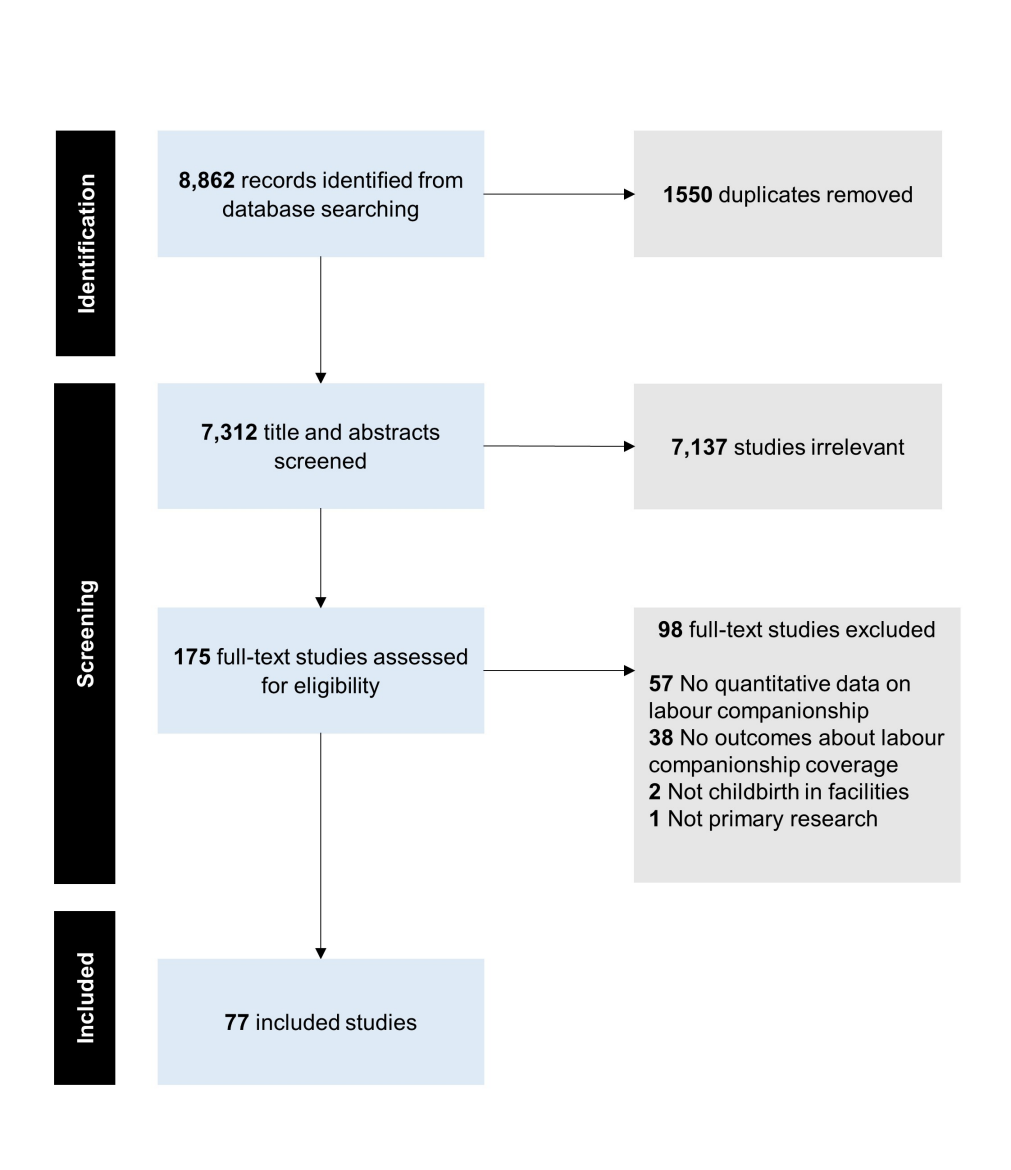
PRISMA flowchart depicting search and selection process.

We identified 8,862 citations from the database searches, and included 77 studies that were published between 2010 and 2021 in English, Mandarin, Portuguese, and Spanish (Fig 1. PRISMA flowchart).

### Characteristics of included studies

Table 1 reports a summary of the characteristics of included studies; S1 Table reports characteristics of each included study. In summary, the 77 included studies were conducted in 27 countries. This included 25 studies in the African region (9 countries: Ethiopia [11–17], Tanzania [18–21], Kenya [22–25], Ghana [21, 26, 27], Mozambique [28–30], Nigeria [27, 31, 32], Rwanda [33, 34], Guinea [27], and South Africa [35]); 30 studies in the Americas region (3 countries: Brazil [36–56], United States of America [57–63], and Chile [64, 65]); three studies in the Eastern Mediterranean region (2 countries: Iran [66, 67], and Saudi Arabia [68]); six studies in the European region (5 countries: Spain [69, 70], Austria [71], Germany [72], Italy [73], and Slovenia [74]; twelve studies in the Southeast Asian region (7 countries: Bangladesh [21, 75, 76], Nepal [77–79], India [80, 81], Myanmar [27], Pakistan [82], Sri Lanka [83], and Thailand-Myanmar border [84]); and three studies in the Western Pacific region (1 country: China [85–87] (note: two multi-country studies were conducted in countries in the African and Southeast Asian regions [21, 27]). Most studies were conducted in low- or middle-income countries: 17.9% (14/78) in low-income [11–17, 19, 27–30, 33, 34], 25.6% (20/78) in lower-middle income [18, 20–27, 31, 32, 75–83], 35.9% (28/78) in upper-middle income countries [35–56, 66, 67, 84–87], and 20.5% (16/78) in high income countries [57–65, 68–74] (note: one multi-country study was conducted in both low- and lower-middle income countries [27].

**Table 1:** Characteristics of included studies.

Characteristics	n (%) n = 77 studies
**Region^1^ (n = 79)**	
African	25 (31.6%)
Americas	30 (38.0%)
Eastern Mediterranean	3 (3.8%)
European	6 (7.6%)
Southeast Asian	12 (15.2%)
Western Pacific	3 (3.8%)
**Country-income level^2^ (n = 78)**	
Low	14 (17.9%)
Lower-middle	20 (25.6%)
Upper-middle	28 (35.9%)
High	16 (20.5%)
**Study setting**	
Urban	39 (50.6%)
Rural	11 (14.3%)
Both urban and rural	11 (14.3%)
Not specified	16 (20.8%)
**Type of recruitment**	
Facility-based	62 (80.5%)
Population-based	15 (19.5%)
**Type of health facility^3^**	
Public hospital	32 (41.6%)
Non-public hospital	3 (3.9%)
Mixed public and non-public hospital	21 (27.3%)
Population-based	15 (19.5%)
Not specified	6 (7.8%)
**Timing of coverage measurement**	
Before 2010	8 (10.4%)
2010–2014	23 (29.9%)
2015–2019	36 (46.8%)
2020–2022	8 (10.4%)
Not specified	2 (2.6%)
**Duration of data collection**	
<6 months	43 (55.8%)
6–12 months	13 (16.9%)
≥12 months	14 (18.2%)
Not specified	7 (9.1%)
**Study design^4^**	
Cross-sectional	56 (72.7%)
Randomised or non-randomised trial	8 (10.4%)
Mixed-methods	7 (9.1%)
Pre-post test	4 (5.2%)
Retrospective or prospective cohort	2 (2.6%)
**Sample size**	
0–499	36 (46.8%)
500–999	19 (24.7%)
1000–4999	15 (19.5%)
≥5000	5 (6.5%)
Not specified	2 (2.6%)
**Language of publication**	
English	69 (89.6%)
Portuguese	6 (7.8%)
Chinese	1 (1.3%)
Spanish	1 (1.3%)

^1^ Two multi-country studies were conducted in countries in the African and Southeast Asian regions (Balde, 2020; Manu, 2021).

^2^ At the time of publication; one multi-country study was conducted in both low- and lower-middle income countries (Balde, 2020).

^3^ Non-public hospitals include private and charity hospitals.

^4^ Data from trials and pre- and post-test studies were only included from the baseline or control group

Most studies used health facility-based recruitment (80.5%, 62/77 studies), and the remaining studies used population-based recruitment (19.5%, 15/77 studies). There was variation in the types of health facilities, including public hospitals (41.6%, 32/77 studies), non-public hospitals such as private or charity hospitals (3.9%, 3/77 studies), and mixed public and non-public hospitals (27.3%, 21/77 studies). Most studies used a cross-sectional design (72.7%, 56/77 studies), followed by randomised or non-randomised trial (10.4%, 8/77 studies), mixed-methods design (9.1%, 7/77 studies), pre-post test (5.2%, 4/77 studies), and cohort design (2.6%, 2/77 studies).

### Labour companionship coverage and characteristics

Table 2 provides a summary of labour companionship coverage and characteristics, and S2 Table reports labour companionship coverage and characteristics for each study. There was wide variation in the coverage of labour companionship, and where coverage was reported in multi-country studies, we report separately by country (82 data points from 77 studies). Almost one-third of studies reported coverage of labour companionship below 40% (**0–19.9% coverage**: 22.0%, 18/82 studies; **20–39.9% coverage**: 8.5%, 7/82 studies). Another one-third of studies reported coverage of labour companionship between 40–80% (**40–59.9% coverage**: 17.1%, 14/82 studies; **60–79.9% coverage**: 13.4%, 11/82 studies). Thirty-nine percent of studies (32/82 studies) reported coverage of labour companionship of **80–100% coverage**.

**Table 2:** Labour companionship coverage and characteristics.

Characteristics	n (%) n = 77 studies	References
**Coverage of labour companionship^1^ (n = 82)**		
0.0–19.9%	18 (22.0%)	[12–14, 21, 28, 32, 33, 35, 43, 55, 60, 66, 77, 78, 80, 83, 85]
20.0–39.9%	7 (8.5%)	[11, 22, 29, 34, 54, 68, 86]
40.0–59.9%	14 (17.1%)	[16, 18–20, 26, 30, 42, 46, 56, 63, 67, 73, 76, 81]
60.0–79.9%	11 (13.4%)	[17, 24, 25, 47, 50, 51, 61, 64, 65, 71, 79]
80.0–100.0%	32 (39.0%)	[15, 21, 23, 27, 31, 36–41, 44, 45, 48, 49, 52, 53, 57–59, 62, 69, 70, 72, 74, 75, 82, 84, 87]
**Woman allowed companion of her choice^2^**		
Yes	22 (28.6%)	[13, 14, 16, 22, 24, 26, 28, 35, 37, 41, 45, 48, 50, 51, 53, 54, 65, 70, 71, 76, 82, 85]
No	2 (2.6%)	[32, 80]
Did not specify	53 (68.8%)	[11, 12, 15, 17–21, 23, 25, 27, 29–31, 33, 34, 36, 38–40, 42–44, 46, 47, 49, 52, 55–64, 66–69, 72–75, 77–79, 81, 83, 84, 86, 87]
**Type of companion present during labour and/or birth^3^**		
Husband or partner	29 (37.7%)	[13, 19, 20, 24, 25, 27, 28, 30, 33–36, 41, 44, 45, 50, 52, 54, 58, 59, 61, 62, 71, 72, 74, 76, 79, 84, 85]
Any family member or friend (gender not specified)	25 (32.5%)	[16, 17, 19, 20, 23–25, 27, 30, 31, 37, 41, 44, 45, 52–54, 61, 62, 72, 74, 75, 78, 85, 87]
Doula	12 (15.6%)	[27, 37, 44, 50, 53, 60–62, 66, 74, 86, 87]
Family member or friend (female only)	10 (13.0%)	[13, 24, 25, 30, 45, 50, 54, 71, 76, 84]
Traditional birth attendant	4 (5.2%)	[19, 20, 27, 84]
Did not specify	36 (46.8%)	[11, 12, 14, 15, 18, 21, 22, 26, 29, 32, 38–40, 42, 43, 46–49, 51, 55–57, 63–65, 67–70, 73, 77, 80–83]
**Timing companionship allowed for women with vaginal birth^2^^,^^4^**		
During labour only	15 (19.5%)	[13, 24, 26, 31, 34, 39, 41, 56, 57, 60, 61, 65, 67, 78, 85]
During birth only	11 (14.3%)	[17, 21, 35, 53, 55, 58, 59, 73, 79, 83, 86]
During labour and birth	25 (32.5%)	[12, 15, 16, 19, 20, 22, 23, 28, 29, 33, 37, 40, 42, 46, 51, 52, 62–64, 70, 71, 76, 77, 82, 84]
During labour, birth and postnatal	8 (10.4%)	[25, 43–45, 49, 50, 54, 81]
Companion not allowed	2 (2.6%)	[32, 80]
Did not specify	16 (20.8%)	[11, 14, 18, 27, 30, 36, 38, 47, 48, 66, 68, 69, 72, 74, 75, 87]
**Companion allowed during caesarean birth^2^^,^^4^ (n = 20)**		
Yes	11 (55.0%)	[16, 23, 37, 42, 44, 59, 62, 64, 71–73]
No	4 (20.0%)	[13, 31, 57, 65]
Did not specify	5 (25.0%)	[29, 33, 47, 49, 69]

^1^ Where coverage is reported in multi-country studies, we report coverage separately by country (Balde, 2020; Manu,2021).

^2^ Allowed by the health facility or health worker

^3^ Total >77 studies, as support from more than one type of companion was recorded in some studies.

^4^ 20/77 studies included women with CS as participants

Most studies did not specify if a woman was allowed to choose the companion who supported her (68.8%, 53/77 studies), and 28.6% of studies (22/77 studies) reported that a woman was allowed to choose her companion.

Women in the included studies were supported by a variety of companions; 41 of 77 studies (53.2%) reported the type of companion at the woman-level. Given heterogeneity in outcome reporting, we summarise the most common types of companions, based on what person or people were reported to have acted as companions at the individual woman-level. Women in the same health facility and/or study may be supported by different companions (e.g., some supported by a partner or husband, some by a friend), and some women were supported by more than one companion. Partners or husbands were the most common type of companion (37.7%, 29/77 studies), followed by any family member or friend (gender not specified) (32.5%, 25/77 studies), doula (15.6%, 12/77 studies), family member or friend (female only) (13.0%, 10/77 studies), and traditional birth attendants (5.2%, 4/77 studies).

There was variation in which time periods health facilities or health workers allowed labour companions to support women with vaginal birth: during labour only (19.5%, 15/77), during birth only (14.3%, 11/77, during labour and birth (32.5%, 25/77), and throughout labour, birth and postnatal (10.4%, 8/77). Eighteen studies (23.4%) did not specify which time periods companions could be present or did not allow companionship. While most studies only included women who had vaginal birth at the most recent pregnancy (74%, 57/77), 20 studies (26.0%) included women who had caesarean births at the most recent pregnancy, and of these, 11 studies (55.0%) were in health facilities that allowed companions to be present during the caesarean birth.

### Coverage of labour companionship in a network of sentinel sites in Latin America

A total of 120,581 women gave birth at the nine sentinel sites in five countries during the study period (April 2018 to April 2022). Table 3 reports whether the woman had a companion during labour only, during birth only, during labour and birth, no companion at any time, and missing data on one or more time periods. A total of 59.0% of women (n = 71,123/120,581) had no companion present at any time, ranging from 14.9% to 93.8% across hospitals. A total of 10.7% (n = 12,958/120,581) of women had a companion during both labour and birth, ranging from 0.2% to 44.3% across hospitals. 6.8% of women (n = 8,159/120,581) had a companion present during labour only, and 0.1% of women (n = 159/120,581) had a companion present during birth only. Data on the presence of a companion were missing from either labour, birth, or both periods for 23.4% of women (n = 28,182/120,581).

**Table 3:** Coverage of labour companionship from SIP sentinel data in 9 Latin American hospitals^1^.

Country and site	Total women giving birth	Women with companion during labour only		Women with companion during birth only		Women with companion during labour and birth		Women with no companion at any time		Missing	
	n	n	%	n	%	n	%	n	%	n	%
Bolivia Hospital 1	7,440	6,174	83.0%	3	0.0%	13	0.2%	1,106	14.9%	144	1.9%
Bolivia Hospital 2	1,142	29	2.5%	0	0.0%	25	2.2%	893	78.2%	195	17.1%
Guatemala Hospital 1	17,334	517	3.0%	42	0.2%	957	5.5%	10,885	62.8%	4,933	28.5%
Honduras Hospital 1	24,793	972	3.9%	66	0.3%	2,876	11.6%	12,052	48.6%	8,827	35.6%
Honduras Hospital 2	8,096	368	4.5%	18	0.2%	361	4.5%	5,881	72.6%	1,468	18.1%
Honduras Hospital 3	2,500	15	0.6%	0	0.0%	10	0.4%	2,112	84.5%	363	14.5%
Nicaragua Hospital 1	18,950	7	0.0%	7	0.0%	8,395	44.3%	10,442	55.1%	99	0.5%
Nicaragua Hospital 2	13,889	2	0.0%	2	0.0%	66	0.5%	13,033	93.8%	786	5.7%
Dominican Republic Hospital 1	26,437	75	0.3%	21	0.1%	255	1.0%	14,719	55.7%	11,367	43.0%
All hospitals	120,581	8,159	6.8%	159	0.1%	12,958	10.7%	71,123	59.0%	28,182	23.4%

Table 4 reports the companion’s relationship to the woman. Among women who had a companion present during labour, a family member was the most common companion across all sites (n = 16,517/28,739, 57.5%), followed by a partner or husband (n = 10,326/28,739, 35.9%).

**Table 4:** Labour companion’s relationship to the woman from SIP sentinel data in nine Latin American hospitals.

Country and site	Women with companion during labour^1^ n	Who was the companion		
		Partner/husband n (%)	Family n (%)	Other n (%)
Bolivia Hospital 1	6,280	1488 (23.7%)	4785 (76.2%)	7 (0.1%)
Bolivia Hospital 2	79	44 (55.7%)	27 (34.2%)	8 (10.1%)
Guatemala Hospital 1	2,156	1300 (60.3%)	704 (32.7%)	152 (7.1%)
Honduras Hospital 1	9,524	5331 (56.0%)	3,457 (36.3%)	736 (7.7%)
Honduras Hospital 2	1,213	402 (33.1%)	642 (52.9%)	169 (13.9%)
Honduras Hospital 3	249	36 (14.5%)	141 (56.6%)	72 (28.9%)
Nicaragua Hospital 1	8,404	1522 (18.1%)	6,221 (74.0%)	661 (7.9%)
Nicaragua Hospital 2	71	14 (19.7%)	50 (70.4%)	7 (9.9%)
Dominican Republic Hospital 1	763	189 (24.8%)	490 (64.2%)	84 (11.0%)
All hospitals	28,739	10,326 (35.9%)	16,517 (57.5%)	1,896 (6.6%)

^1^ Total women with companion during labour, regardless if she had a companion during birth, or missing data for birth.

## Discussion

In the absence of global monitoring data about labour companionship, we aimed to identify studies or reports that helped to understand coverage across the globe. We found studies for 27 countries, largely representing one or a few health facilities in each country. Brazil is the country with the highest number of studies with coverage (n = 20) ranging from 4.1 to 100%, which may be explained by researcher interest in documenting the effect of the 2005 national policy introducing labour companionship for all women [42, 44]. We identified wide variation in the coverage of labour companionship across studies included in the scoping review and in the Latin American SIP data. Based on these findings, it appears that labour companionship is still not widely implemented, with over 30% of studies reporting less than 40% coverage and over 60% reporting less than 80% coverage. Efforts are therefore urgently needed to discuss at the national and sub-national levels how to ensure policies are available on labour companionship and ensure discussions with women, communities, health workers and health programme managers to address implementation barriers.

In settings where some but not all women have a labour companion, more work is needed to understand who is missing this support, in order to understand equity implications, as well as how quality of care may vary at the point of care for different women. For example, if rich, white, or educated women have better access to support from a labour companion, compared to poor, Indigenous, Black, migrant, or less educated women giving birth in the same health facilities or in different health facilities, then substantial effort will be needed to reduce inequities and ensure non-discriminatory care. The equity issues related to who benefits and who misses out on support from a labour companion are well documented in existing research literature. A study conducted by Balde and colleagues (2020) in Ghana, Guinea, Myanmar and Nigeria found that younger women (<20 years), unmarried women, and women with no or limited education were less likely than their counterparts to receive support from a labour companion [27]. Afulani and colleagues (2018) found similar results: women who were literate, wealthy, and employed were more likely to benefit from labour companionship [25]. These inequities in who receives support from a labour companion have also led to the development of critical programmes to support socially marginalised women, for example via community-based doula support for Indigenous women in Canada [88], Black and Latinx women in New York [89], and women from migrant and refugee backgrounds in Australia and the United Kingdom [90, 91].

Only 28.6% of studies included in the scoping review reported that women were allowed to choose their companion. WHO recommends that women are offered a “companion of choice” [3, 7] and grounds this recommendation within human rights principles to ensure women’s autonomy, agency, and choice about their companion. More effort is needed to create enabling environments within health facilities to support women to make informed choices about companionship, to support families and other community members to understand the importance of labour companionship, and to orient health workers to the benefits of companionship. In addition to fostering enabling environments, women should receive information, education, and a means to make and implement these choices effectively [7], in a way that ensures no undue influence from a partner, family member, or others. In some settings and for some women, having a husband or partner as a labour companion may be helpful and appropriate for parental bonding. However, in contexts where the presence of men is less appropriate (e.g., where pregnancy and birth are considered ‘women’s business’) [92], female family members or friends can act as labour companions.

Across the studies included in the scoping review and analysis of SIP sentinel data, we found that companionship for women with vaginal birth was not typically across the continuum of labour and childbirth. We identified two threats that may help to explain these variations in continuous support. First, in some settings, companions are allowed during labour, but must leave for the birth, which may be due to space constraints in settings where women give birth in a birthing room separate from the labour ward, or because the support provided by companions is not valued by health management or health workers. Second, in some settings (particularly Latin America), companions may be allowed to be present during the birth only, but not during labour, which may be due to busy labour wards with limited space and lack of privacy. To address both threats to continuous support, some restructuring to the labour and birthing spaces may be needed. For example, a study implementing labour companionship in Lebanon, Syria, and Egypt addressed space constraints by making adjustments to the labour rooms to include chairs for companions, curtains around beds, and access to toilet facilities [92].

As little is known world-wide about coverage of labour companion, the inclusion of data from SIP record is a good example of how health facilities can capture and include information in routine systems so that uptake can be monitored. If needed, additional analysis could be done to determine equity gaps, and identify areas for improvement. Monitoring labour companionship at the national level is critical to identify areas for improvement, and particularly to generate data in settings where labour companionship is not yet a common practice. Nonetheless, the high amount of missing data confirms that the problems of data quality across routine records and health management information systems [93] and may question confidence in the data.

Reporting of the characteristics of labour companionship was also inconsistent across studies, with many not specifying clearly who the companion was. Given the lack of available information on coverage and how companionship is implemented, it would be helpful if studies could report on certain core information, such as was the woman allowed to choose the companion, who was the companion, and across what periods they were present.

## Strengths and limitations

This is the first global scoping review to better understand the coverage and characteristics of labour companionship and the results can be used in conjunction with WHO recommendations to justify prioritisation of global monitoring, country dialogue to address policy changes and uptake and implementation research to identify models to be scaled up. We used robust scoping review methods, along with analysis of sentinel data to assess coverage of labour companionship in practice to identify opportunities for improvement. Our approach has some limitations. First, we planned to use the WHO reproductive, maternal, newborn, child and adolescent health policy survey (2018) to assess the extent to which labour companionship was included in national policies for maternal health and to analyse the content of policy documents to explore language on labour companionship in national policies or guidelines. However, the related documents uploaded to the survey were incomplete, and as a result, we dropped this component from our review. As mentioned, there was also missing data in SIP for the labour companionship indicator, meaning that labour companionship coverage may be underestimated in our analysis.

### Implications for policy, practice, monitoring and research

Given that the health and well-being benefits of labour companionship are well understood [1], increased efforts need to be undertaken to improve coverage in an equitable manner, and across labour and childbirth periods. Concerted efforts are needed to engage with women, communities, health workers, health managers, and policy-makers to establish policies, address implementation barriers, and integrate data on coverage into existing perinatal records and quality processes to ensure that all women have access to labour companionship across the continuum of labour and childbirth. National and sub-national policies and guidelines may need revision to align with the WHO recommendations on companions of choice for women [3, 7]. Implementation research approaches can help to translate these policies into health facility-level policy and practice, while ensuring that local context and values are accounted for. Existing reviews provide overviews of factors that may affect implementation of labour companionship, noting that while acceptability of labour companion support among women and families is often high, constraints around feasibility can hamper implementation efforts [2, 94]. These constraints include existing policies disallowing or limiting labour companion support, physical space constraints, perceived risk of infection, and perceived need for training of labour companions before birth [2, 94]. Moreover, women may not be aware of their rights to receive quality maternity care and the importance of labour companionship, which can be addressed through antenatal education programmes or in community discussions [95]. Existing implementation research projects and programmes have addressed context-specific constraints to introduce labour companionship in different settings, for example, by using participatory approaches with health workers and with community stakeholders, making physical changes to the labour ward environment, and orienting health workers, women, and potential companions about how to best support women [92, 96, 97].

Additionally, labour companionship can be implemented into other research studies and implementation activities aiming to improve the quality of maternity care, or quality improvement studies, as a key intervention to improve birth experiences and respectful maternity care [27]. For example, the new WHO Labour Care Guide includes assessment and monitoring of supportive care practices including labour companionship [98], and implementation research to introduce the WHO Labour Care Guide can also include efforts to improve labour companionship.

Perinatal records and routing recording systems can include collection on if a companion was present so that coverage can be monitored. This may also allow for better understanding at global level about the uptake of this important intervention.

Finally, our scoping review identified a wide range of definitions, outcomes, and indicators used to describe support by a labour companion. Some terminology was vague (e.g., ‘supportive care received’), which limited comparisons. We suggest that researchers and programmers consider the following when implementing and reporting labour companionship in order to ensure the generation of actionable evidence:

1. Reporting companionship separately for labour, birth, and postnatal periods, or as a composite across this continuum to ensure that measurement reflects the complexities of implementation;
2. Consider how labour companionship can be adapted for women experiencing both pre-labour and in-labour caesarean sections to ensure that all women have access to support, regardless of mode of birth;
3. Using the terminology of ‘labour companion’ preferentially over ‘supportive care’, which could reflect ‘supportive care’ by a health worker or labour companion;
4. Reporting whether it was a ‘companion of choice’; and
5. Reporting both whether a woman is ‘offered the option to experience companionship’ and whether she has ‘received support from a labour companion,’ to reflect the nuance that not all women will want a lay labour companion.

### Conclusions

We identified wide variations in coverage, highlighting the urgent need for substantial efforts to ensure that all women have the opportunity to be supported by a labour companion. Efforts to improve global maternal health to focus on improving birth experiences and respectful maternity care should consider how to change policy and practice to enable support from labour companions across the continuum of labour and childbirth.

## Supporting information

S1 Appendix PRISMA-ScR checklist. (PDF)

S2 Appendix Search strategies. (PDF)

S1 Table Characteristics of included studies–study level. (PDF)

S2 Table Labour companionship coverage and characteristics–study level. (PDF)
