# Supplementary material for: From global recommendations to (in)action: A scoping review of the coverage of companion of choice for women during labour and birth
Source: PLOS Glob Public Health. 2023 Feb 1;3(2):e0001476. doi: 10.1371/journal.pgph.0001476 (PMC10021298; doi:10.1371/journal.pgph.0001476)
Supplement: S2 Appendix — (PDF) [file pgph.0001476.s002.pdf]

S2 Appendix. Search strategies

Ovid MEDLINE

#	Query	Results from 14 Dec 2021
1	Perinatal Care/	5,110
2	Obstetric Nursing/	3,051
3	Delivery, Obstetric/	31,567
4	Labor, Obstetric/	30,148
5	Parturition/	11,421
6	Home Childbirth/	2,899
7	Natural Childbirth/	2,458
8	or/1-7	76,238
9	Social Support/	75,679
10	8 and 9	881
11	Doulas/	176
12	(doula or doulas or obstetric nursing).ti,ab,kw,kf.	542
13	((childbirth* or birth* or labor or laboring or labour or labouring or intrapartum) adj6 (support* or companion* or coach*)).ti,ab,kw,kf.	4,692
14	(((presence or present or attend* or accompan*) adj3 (family member* or friend* or spouse* or partner* or unskilled)) and (childbirth* or birth* or labor or labour)).ti,ab,kw,kf.	328
15	(((presence or present or attend* or accompan*) adj3 (midwife or midwives or midwifery or nurse)) and (childbirth* or birth* or labor or labour)).ti,ab,kw,kf.	525
16	or/11-15	5,838
17	10 or 16	6,425
18	limit 17 to yr="2010 -Current"	3,970

CINAHL, Ebsco (14 Dec 2021)

#	Query
S 1	(MH "Prenatal Care")
S 2	(MH "Obstetric Nursing")
S 3	(MH "Delivery, Obstetric")
S 4	(MH "Labor")
S 5	(MH "Childbirth+")
S 6	S1 OR S2 OR S3 OR S4 OR S5
S 7	(MH "Support, Psychosocial")
S 8	(MH "Caregiver Support")
S 9	S7 OR S8
S 10	S6 AND S9
S 11	(MH "Doulas")
S 12	(MH "Labor Support")
S 13	TI (doula or doulas or "obstetric nursing") OR AB (doula or doulas or "obstetric nursing")
S 14	TI ((childbirth* or birth* or labor or laboring or labour or labouring or intrapartum) N6 (support* or companion* or coach*)) OR AB ((childbirth* or birth* or labor or laboring or labour or labouring or intrapartum) N6 (support* or companion* or coach*))
S 15	TI (((presence or present or attend* or accompan*) N3 ("family member" or "family members" or friend* or spouse* or partner* or unskilled)) and (childbirth* or birth* or labor or labour)) OR AB (((presence or present or attend* or accompan*) N3 ("family member" or "family members" or friend* or spouse* or partner* or unskilled)) and (childbirth* or birth* or labor or labour))
S 16	TI ((presence or present or attend* or accompan*) N3 (midwife or midwives or midwifery or nurse) and (childbirth* or birth* or labor or labour)) OR AB ((presence or present or attend* or accompan*) N3 (midwife or midwives or midwifery or nurse) and (childbirth* or birth* or labor or labour))
S 17	S11 OR S12 OR S13 OR S14 OR S15 OR S16
S 18	S10 OR S17
S 19	Limiters - Published Date: 20100101-20221231; Exclude MEDLINE records

Global Health, CAB Direct (14 Dec 2021)

All fields: (care AND support AND childbirth)

OR

All fields: ("kinship network*" AND childbirth) OR

OR

All fields: (doula OR doulas OR “prenatal support” OR “childbirth support” OR “birth support” OR “labor support” OR “labour support” OR “intrapartum support” OR “childbirth companion” OR “childbirth companionship” OR “birth companion” OR “birth companionship” OR “labor companion” OR “labor companionship” OR “labour companion” OR “labour companionship” OR “support during labor” OR “support during labour” OR “support during childbirth” OR “support during birth” OR “support during delivery”)

AND yr:[2010 TO 2021]
