# Supplementary material for: From global recommendations to (in)action: A scoping review of the coverage of companion of choice for women during labour and birth
Source: PLOS Glob Public Health. 2023 Feb 1;3(2):e0001476. doi: 10.1371/journal.pgph.0001476 (PMC10021298; doi:10.1371/journal.pgph.0001476)
Supplement: S1 Table — (PDF) [file pgph.0001476.s003.pdf]

S1 Table. Characteristics of included studies – study level

Authors	Year	Title	Context	Language of publication	Type of recruitment	Type of health facility	Timing of coverage measurement	Duration of data collection	Study design	Sample size
Aduloju	2013	Pain perception among parturients at a University Teaching Hospital, South-Western Nigeria	Ado-Ekiti, Nigeria; 1 health facility	English	Facility-based	Public hospital	January 2009 - December 2010	24 months	Cross-sectional	1012
Afulani	2018	Companionship during facility-based childbirth: results from a mixed-methods study with recently delivered women and providers in Kenya	Western Kenya, Kenya; 8 sub-counties	English	Population-based	Population-based	August 2016 - September 2016	2 months	Cross-sectional	857
Afulani	2019	Can an integrated obstetric emergency simulation training improve respectful maternity care? Results from a pilot study in Ghana	East Mamprusi District, Ghana; 5 health facilities	English	Facility-based	Mixed public and private hospital	March 2017 - April 2017	2 months	Pre-post study	215
Agha	2019	Quality of labor and birth care in Sindh Province, Pakistan: Findings from direct observations at health facilities	Sindh, Pakistan; 47 health facilities	English	Facility-based	Mixed public and private hospital	April 2014 - June 2014	3 months	Cross-sectional	310
Al-Mandeel	2013	Saudi womens acceptance and attitudes towards companion support during labor: should we implement an antenatal awareness program?	Riyadh, Saudi Arabia; 3 health facilities	English	Facility-based	Public hospital	April 2010 - June 2010	3 months	Prospective cohort	402
Balde	2020	Labour companionship and women's experiences of mistreatment during childbirth: results from a multi-country community-based survey	Ghana, Guinea, Myanmar, Nigeria; 3 health facilities per country	English	Facility-based	Public hospital	Ghana: August 2017 – January 2018<br>Guinea: July 2017 – October 2017<br>Myanmar: June – September 2017	Ghana: 6 months<br>Guinea: 4 months<br>Myanmar: 4 months	Cross-sectional	Total sample size across four countries: 2672

Authors	Year	Title	Context	Language of publication	Type of recruitment	Type of health facility	Timing of coverage measurement	Duration of data collection	Study design	Sample size
							Nigeria: September 2016 – February 2017	Nigeria: 6 months		
Baldisserotto	2016	Good practices according to WHO's recommendation for normal labor and birth and women's assessment of the care received: the "birth in Brazil" national research study, 2011/2012	Southeast region, Brazil; 89 health facilities	English	Facility-based	Mixed public and private hospital	February 2011 – October 2012	21 months	Cross-sectional	4102
BarrosGuida	2017	Compliance of nursing care practices with technical recommendations for normal birth	Rio de Janeiro city, Brazil; 2 health facilities	English	Facility-based	Public hospital	April 2015 – August 2015	5 months	Cross-sectional	520
Berhane	2019	Quality of basic emergency obstetric and newborn care (BEmONC) services from patients' perspective in Adigrat town, Eastern zone of Tigray, Ethiopia. 2017: a cross sectional study	Eastern zone of Tigray, Ethiopia; 3 health facilities	English	Facility-based	Mixed public and private hospital	2017	Study did not specify	Cross-sectional	398
BeyeneGetahun	2020	Utilization of companionship during delivery and associated factors among women who gave birth at Arba Minch town public health facilities, southern Ethiopia	Arbaminch town, Ethiopia; 3 health facilities	English	Facility-based	Mixed public and private hospital	October 2019 – November 2019	2 months	Cross-sectional	407
Bezerra	2019	Perinatal care in a Northeastern Brazilian State: structure, work processes, and evaluation of the components of essential newborn care	State of Sergipe, Brazil; 11 health facilities	English	Facility-based	Mixed public and private hospital	June 2015 - April 2016	11 months	Cross-sectional	768
Bharti	2021	Establishing the practice of birth companion in labour ward of a tertiary	New Delhi, India; 1 health facility	English	Facility-based	Public hospital	January 2019	Study did not specify	Pre-post study	Study did not specify

Authors	Year	Title	Context	Language of publication	Type of recruitment	Type of health facility	Timing of coverage measurement	Duration of data collection	Study design	Sample size
		care centre in India-a quality improvement initiative								
Binfa	2016	Assessment of the implementation of the model of integrated and humanised midwifery health services in Chile	North, central, metropolitan and southern areas, Chile; 9 health facilities	English	Facility-based	Public hospital	May 2013 - December 2013	8 months	Cross-sectional	1729
Blanc	2016	Assessing the validity of indicators of the quality of maternal and newborn health care in Kenya	Kisumu and Kiambu district, Kenya; 2 health facilities	English	Facility-based	Public hospital	July 2013 - September 2013	3 months	Cross-sectional	662
Cederfeldt	2016	Quality of intra-partum care at a university hospital in Nepal: a prospective cross-sectional survey	Nepal; 1 health facility	English	Facility-based	Public hospital	November 2013	3 weeks	Cross-sectional	164
Chaote	2021	Birth companionship in a government health system: a pilot study in Kigoma, Tanzaia	Kigoma region, Tanzania; 9 health facilities	English	Facility-based	Mixed public and private hospital	October 2017	1 month	Randomised or non-randomised trial	Study did not specify
Cheung	2011	Clinical outcomes of the first midwife-led normal birth unit in China: a retrospective cohort study	Hangzhou city, China; 1 health facility	English	Facility-based	Public hospital	March 2008 - September 2008	7 months	Randomised or non-randomised trial	Total sample: 452 Control group (data used): 226
Cortes	2018	Implementation of evidence-based practices in normal delivery care	Macapá, Brazil; 1 health facility	English	Facility-based	Public hospital	July 2015 - March 2016	9 months	Pre-post study	140

Authors	Year	Title	Context	Language of publication	Type of recruitment	Type of health facility	Timing of coverage measurement	Duration of data collection	Study design	Sample size
daMattaMachadoFernandes	2021	Brazilian women's use of evidence-based practices in childbirth after participating in the Senses of Birth intervention: a mixed- methods study	Belo Horizonte, Rio de Janeiro, Niterol, Ceilandia and Brasilia, Brazil; 3 states	English	Population-based	Population-based	March 2015 - March 2016	13 months	Randomised or non-randomised trial	555
Declercq	2014	Major survey findings of Listening to Mothers III: pregnancy and birth	USA; nationwide	English	Population-based	Population-based	July 2011 - June 2012	12 months	Cross-sectional	2400
deMouraAlves	2019	Contribuições da enfermagem obstétrica para as boas práticas no trabalho de parto e parto vaginal	The state of Goiás, Brazil; 1 health facility	Portuguese	Facility-based	Public hospital	January 2016 – December 2016	12 months	Cross-sectional	475
Dim	2011	Labor support: an overlooked maternal health need in Enugu, south-eastern Nigeria	Enugu, Nigeria; 1 health facility	English	Facility-based	Public hospital	January 2006 - August 2006	8 months	Cross-sectional	395
Diniz	2014	Implementação da presença de acompanhantes durante a internação para o parto: dados da pesquisa nacional Nascer no Brasil	North, northeast, southeast, central and south areas, Brazil; 266 health facilities	Portuguese	Facility-based	Mixed public and private hospital	February 2011 - October 2012	12 months	Cross-sectional	23879
Donati	2021	Childbirth Care among SARS-CoV-2 Positive Women in Italy	Italy; nationwide	English	Population-based	Population-based	February 2020 - July 2020	6 months	Prospective cohort study	525
dosSantosMoura	2020	Análise de práticas na assistência ao parto e pós-parto hospitalar	Pernambuco State, Brazil; 1 health facility	Portuguese	Facility-based	Public hospital	September 2018 - February 2019	6 months	Cross-sectional	335
Drysdale	2021	Father involvement, maternal depression and child nutritional outcomes in Soweto, South Africa	Soweto, South Africa; 1 health facility	English	Facility-based	Public hospital	March 2019 - August 2020	18 months	Randomised or non-randomised trial	212
Dynes	2019	Client and provider factors associated with companionship during labor and birth in Kigoma Region, Tanzania	Kigoma region, Tanzania; 61 health facilities	English	Facility-based	Mixed public and	April 2016 - July 2016	4 months	Cross-sectional	935

Authors	Year	Title	Context	Language of publication	Type of recruitment	Type of health facility	Timing of coverage measurement	Duration of data collection	Study design	Sample size
						private hospital				
Dynes	2018	Patient and provider determinants for receipt of three dimensions of respectful maternity care in Kigoma Region, Tanzania- April-July, 2016	Kigoma region, Tanzania; 61 health facilities	English	Facility-based	Mixed public and private hospital	April 2016 - July 2016	4 months	Cross-sectional	935
Galle	2019	Disrespect and abuse during facility-based childbirth in southern Mozambique: a cross-sectional study	Manhiça and Marracuene, Maputo Province, Mozambique; 3 health facilities	English	Facility-based	Public hospital	April 2018 - June 2018	3 months	Cross-sectional	520
Giordano	2019	The role of the respectful maternity care model in São Paulo,Brazil: a cross-sectional study	São Paulo, Brazil; 1 city	English	Population-based	Population-based	January 2014 - December 2017	36 months	Cross-sectional	580
GoncalvesAde	2015	The companion in the obstetrics centre of a university hospital in southern Brazil	Porto Alegre, Brazil; 1 health facility	English	Facility-based	Public hospital	August 2012 - November 2012	4 months	Cross-sectional	385
Gutiérrez-Martín	2020	Estudio prospectivo de la contribución de la matrona en la atención al parto normal	West Valladolid health area, Spain; 1 health facility	Spanish	Facility-based	Public hospital	February 2017 - June 2017	5 months	Prospective cohort Study	368
Hajizadeh	2020	Prevalence and predictors of perceived disrespectful maternity care in postpartum Iranian women: a cross-sectional study	Tabriz city, Iran; 6 health facilities	English	Facility-based	Mixed public and private hospital	June 2019 - September 2019	4 months	Cross-sectional	334
He	2012	Current situation of doula delivery service in Shanghai city	Shanghai, China; 24 health facilities	Chinese	Facility-based	Not specified	2009 - 2010	Study did not specify	Cross-sectional	82745

Authors	Year	Title	Context	Language of publication	Type of recruitment	Type of health facility	Timing of coverage measurement	Duration of data collection	Study design	Sample size
Hoogenboom	2015	Quality of intrapartum care by skilled birth attendants in a refugee clinic on the Thai-Myanmar border: a survey using WHO Safe Motherhood Needs Assessment	Mae La refugee camp, Thailand - Myanmar border; 1 health facility	English	Facility-based	Not specified	November 2008 – December 2008	2 months	Cross-sectional	20
HunieAsratie	2021	Labor companionship in labor and delivery at Debremarkos Town Public Health Facilities: magnitude and associated factors	East Gojam, Ethiopia; 4 health facilities	English	Facility-based	Mixed public and private hospital	February 2021 - March 2021	2 months	Cross-sectional	548
Kalisa	2016	Birth preparedness, complication readiness and male partner involvement for obstetric emergencies in rural Rwanda	Musanze district, Rwanda; 1 health facility	English	Facility-based	Public hospital	July 2015 - Novermber 2015	5 months	Cross-sectional	350
Kc	2020	Coverage, associated factors, and impact of companionship during labor: a large-scale observational study in six hospitals in Nepal	Nepal Flatlands (east to west), Nepal; 6 health facilities	English	Facility-based	Public hospital	July 2018 - August 2018	2 months	Cross-sectional	53872
Kerebih	2020	Quality of intrapartum care at health centers in Jabi Tehinan district, North West Ethiopia: clients' perspective	Jabi Tehinan district, Ethiopia; 41 kebeles	English	Population-based	Population-based	August 2018 - September 2018	2 months	Cross-sectional	378
Khalife-Ghaderi	2021	Examining the experience of childbirth and its predictors among women who have recently given birth	Khaf County, Iran; 1 health facility	English	Facility-based	Non public hospital	August 2018 - November 2018	4 months	Cross-sectional	225
Kiti	2021	Continuous labor support and person-centered maternity Care: a cross-sectional study with women in rural Kenya	Migori County, Kenya; 80 health facilities	English	Facility-based	Not specified	August 2016 - September 2016	2 months	Cross-sectional	865
Kozhimannil	2014	Potential benefits of increased access to doula support during childbirth	USA; nationwide	English	Population-based	Population-based	July 2011 - June 2012	12 months	Cross-sectional	2400

Authors	Year	Title	Context	Language of publication	Type of recruitment	Type of health facility	Timing of coverage measurement	Duration of data collection	Study design	Sample size
Leal	2019	Progress in childbirth care in Brazil: preliminary results of two evaluation studies	North, northeast, southeast, central and south areas, Brazil; 266 health facilities	English	Facility-based	Mixed public and private hospital	2017	Study did not specify	Cross-sectional	13468
Liu	2021	Unexpected changes in birth experiences during the COVID-19 pandemic: Implications for maternal mental health	USA; nationwide	English	Population-based	Population-based	May 2020 - May 2021	13 months	Cross-sectional	506
Liu	2021	Women's experience and satisfaction with midwife-led maternity care: a cross sectional survey in China	Shanghai, China; 1 health facility	English	Facility-based	Public hospital	March 2019 - June 2019	4 months	Cross-sectional	4192
Lobo	2010	Maternal and perinatal outcomes of an alongside hospital birth center in the city of São Paulo, Brazil	Itaim Paulista district, Brazil; 1 health facility	English	Facility-based	Public hospital	2003-2006	36 months	Retrospective cohort study	991
LohandosSantos	2017	Care practices in normal birth: residence type formation	Municipality of Rio de Janeiro, Brazil; 1 health facility	English	Facility-based	Public hospital	March 2012 - February 2014	24 months	Cross-sectional	827
Lopes	2021	Women's satisfaction with childbirth in a public hospital in Brazil	São Paulo, Brazil; 1 health facility	English	Facility-based	Public hospital	Study did not specify	Study did not specify	Prospective cohort study	372
MachadodosAnjos	2019	Presença do acompanhante durante o processo de parturição e nascimento: análise da prática	Porto Alegre, Brazil; 1 health facility	Portuguese	Facility-based	Public hospital	February 2016 - September 2016	8 months	Cross-sectional	586
Maldie	2021	Magnitude and associated factors of disrespect and abusive care among laboring mothers at public health facilities in Borena District, South Wollo, Ethiopia	Borena district, South Wollo, Ethiopia; 5 health facilities	English	Facility-based	Mixed public and private hospital	January 2020 - March 2020	3 months	Cross-sectional	369

Authors	Year	Title	Context	Language of publication	Type of recruitment	Type of health facility	Timing of coverage measurement	Duration of data collection	Study design	Sample size
Manu	2021	Respectful maternity care delivered within health facilities in Bangladesh, Ghana and Tanzania: a cross-sectional assessment preceding a quality improvement intervention	Kurigram,Lalmonirhat and Gaibandha district, Bangladesh; 15 health facilities<br>Bawku, Kassena-Nankana Eastand Bolgatanga municipalities and Bawku West, Bongo, Kassena-Nankana West and Builsa North districts, Ghana; 16 health facilities<br>Njombe Town Council, Makete, Ludewa and Wanging'ombe district, Tanzania; 12 health facilities	English	Facility-based	Mixed public and private hospital	Bangladesh, Ghana and Tanzania: May 2016 - August 2016	4 months	Cross-sectional	Total sample size across three countries: 641 Bangladesh: 387 Ghana: 134 Tanzania: 120
MartinsFrancoMotta	2016	Implementação da humanização da assistência ao parto natural	Fortaleza, Brazil; 1 health facility	Portuguese	Facility-based	Public hospital	September 2013 - October 2013	2 months	Cross-sectional	51
Menhart	2017	Women's satisfaction with the childbirth experience: a descriptive research	Slovenia; nationwide	English	Population-based	Population-based	February 2017	24 days	Cross-sectional	301
Mocumbi	2019	Mothers' satisfaction with care during facility-based childbirth: a cross-sectional survey in southern Mozambique	Maputo and Gaza Province, Mozambique; 6 districts	English	Population-based	Population-based	June 2016 - October 2016	5 months	Cross-sectional	4358

Authors	Year	Title	Context	Language of publication	Type of recruitment	Type of health facility	Timing of coverage measurement	Duration of data collection	Study design	Sample size
Mollard	2022	Birth satisfaction during the early months of the COVID-19 pandemic in the United States	USA; nationwide	English	Population-based	Population-based	May 2020 – July 2020	3 months	Cross-sectional	747
Mollard	2021	Experiences of Women Who Gave Birth in US Hospitals During the COVID-19 Pandemic	USA; nationwide	English	Population-based	Population-based	May 2020 – July 2020	3 months	Cross-sectional	885
Monguilhott	2018	Nascer no Brasil: the presence of a companion favors the use of best practices in delivery care in the South region of Brazil	South Region, Brazil; 46 health facilities	English	Facility-based	Not specified	February 2011 – August 2011	7 months	Cross-sectional	2070
Mukamurigo	2019	Quality of intrapartum care for healthy women with spontaneous onset of labour in Rwanda: a health facility-based, cross-sectional study	Kigali City and the Northern Province, Rwanda; 10 health facilities	English	Facility-based	Mixed public and private hospital	December 2014 – January 2015	2 months	Cross-sectional	435
Oluoch-Aridi	2021	Examining person-centered maternity care in a peri-urban setting in Embakasi, Nairobi, Kenya	Embakasi within Nairobi City, Kenya; unspecified number of health facilities	English	Facility-based	Mixed public and private hospital	January 2020 – May 2020	5 months	Cross-sectional	307
Pereira dos Santos Moura	2016	Conhecimento e aplicação do direito do acompanhante na gestação e parto	Sergipe, Brazil; 2 health facilities	Portuguese	Facility-based	Non public hospital	April 2015 – May 2015	2 months	Cross-sectional	160
Perkins	2019	Humanised childbirth: the status of emotional support of women in rural Bangladesh	Brahmanbaria district, Bangladesh; 3 upazilas	English	Population-based	Population-based	March 2018 - May 2018	3 months	Cross-sectional	1367
Pervin	2018	Associations between improved care during the second stage of labour and maternal and neonatal health outcomes in a rural hospital in Bangladesh	Matlab sub-district, Bangladesh; 1 health facility	English	Facility-based	Non public hospital	2014-2015	Study did not specify	Pre-post study	523

Authors	Year	Title	Context	Language of publication	Type of recruitment	Type of health facility	Timing of coverage measurement	Duration of data collection	Study design	Sample size
Rishard	2021	Correlation among experience of personcentered maternity care, provision of care and women's satisfaction: Cross sectional study in Colombo, Sri Lanka	Colombo, Sri Lanka; 1 health facility	English	Facility-based	Public hospital	December 2018 - April 2019	5 months	Cross-sectional	400
RodrÃ-guezColl	2021	Women's childbirth satisfaction and obstetric outcomes comparison between two birth hospitals in Barcelona with different level of assistance and complexity	City of Barcelona and Mollet del Vallés, Spain; 2 health facilities	English	Facility-based	Mixed public and private hospital	April 2019 – August 2019	5 months	Cross-sectional	194
Santos	2019	Factors associated with low Apgar in newborns in birth center	Belo Horizonte, Brazil; 1 health facility	English	Facility-based	Public hospital	June 2001 – December 2012	132 months/ 11 years	Retrospective cohort study	9135
Sauls	2010	Promoting a positive childbirth experience for adolescents	Texas, USA; 3 health facilities	English	Facility-based	Not specified	Study did not specify	9 months	Cross-sectional	185
Sheferaw	2017	Respectful maternity care in Ethiopian public health facilities	Tigray, Amhara, Oromia and SNNP regions, Ethiopia; 28 health facilities	English	Facility-based	Mixed public and private hospital	July 2014 - August 2014	2 months	Cross-sectional	240
Simon	2016	Amount, source, and quality of support as predictors of women's birth evaluations	USA; nationwide	English	Population-based	Population-based	2002 and 2006	Study did not specify	Cross-sectional	2765
Singh	2021	Presence of birth companion—a deterrent to disrespectful behaviours towards women during delivery: an exploratory mixed-method study in 18 public hospitals of India	Delhi, Chandigarh, Uttar Pradesh, Rajasthan, Tamil Nadu, Puducherry, Assam, West Bengal, Odisha, Gujarat and Maharashtra, India; 18 health facilities	English	Facility-based	Public hospital	January 2018 – March 2019	15 months	Cross-sectional	1831

Authors	Year	Title	Context	Language of publication	Type of recruitment	Type of health facility	Timing of coverage measurement	Duration of data collection	Study design	Sample size
Spaich	2013	Mode of delivery and its influence on women's satisfaction with childbirth	Germany; unspecified number of health facilities	English	Facility-based	Not specified	May 2010 - September 2011	17 months	Prospective cohort study	335
Stanton	2013	Measuring coverage in MNCH: testing the validity of women's self-report of key maternal and newborn health interventions during the peripartum period in Mozambique	Mozambique; 46 health facilities	English	Facility-based	Mixed public and private hospital	September 2011 - November 2011	2 months	Cross-sectional	440
Tempfer-Bentz	2011	Association of untrained labor companion with operative delivery rate: retrospective study of 2247 women	Vienna, Austria; 1 health facility	English	Facility-based	Public hospital	January 2007 - December 2009	24 months	Retrospective cohort study	2247
Tesfaye	2016	Client satisfaction with delivery care service and associated factors in the public health facilities of Gamo Gofa Zone, Southwest Ethiopia: in a resource limited setting	Gamo Gofa Zone, Ethiopia; 13 health facilities	English	Facility-based	Mixed public and private hospital	December 2013 - January 2014	2 months	Cross-sectional	430
Thapa	2013	Women's autonomy and husbands' involvement in maternal health care in Nepal	Kailali district, Nepal; 4 Village Development Committees (VDC)	English	Population-based	Population-based	September 2011 - November 2011	3 months	Cross-sectional	275
Tomasi	2021	From prenatal care to childbirth: a cross-sectional study on the influence of a companion on good obstetric practices in the Brazilian National Health System in Santa Catarina State, 2019	Santa Catarina State, Brazil; 31 health facilities	English	Facility-based	Public hospital	2019	Study did not specify	Cross-sectional	3580
Vaz	2014	Evaluation of the presence of the companion during birth and the puerperium in a public maternity unit	Curitiba,Paraná, Brazil; 1 Hospital	English	Facility-based	Public hospital	August 2012 - September 2012	2 months	Cross-sectional	105

Authors	Year	Title	Context	Language of publication	Type of recruitment	Type of health facility	Timing of coverage measurement	Duration of data collection	Study design	Sample size
Weeks	2017	Labor and birth care satisfaction associated with medical interventions and accompaniment during labor among Chilean women	Tarapaca, Coquimbo, Valparaıso, Libertador General Bernardo O'Higgins, the Region Metropolitana, Bıobıo, Aysen, and Los Lagos, Chile; 9 health facilities	English	Facility-based	Public hospital	2012 - 2013	Study did not specify	Cross-sectional	1660
