# Supplementary material for: From global recommendations to (in)action: A scoping review of the coverage of companion of choice for women during labour and birth
Source: PLOS Glob Public Health. 2023 Feb 1;3(2):e0001476. doi: 10.1371/journal.pgph.0001476 (PMC10021298; doi:10.1371/journal.pgph.0001476)
Supplement: S2 Table — (PDF) [file pgph.0001476.s004.pdf]

S2 Table. Labour companionship coverage and characteristics – study level

Authors	Year	Coverage of labour companionship	Woman allowed companion of choice	Type of companion present during labour and/or birth						Timing of companionship allowed	Companion allowed during CS
				Any family member or friend (gender not specified)	Family member or friend (female only)	Husband or partner only	Doula	Traditional birth attendant	Study did not specify		
Aduloju	2013	81%	Study did not specify	X						During labour only	No
Afulani	2019	105/215 (48.8%)	Yes						X	During labour only	Study excluded women with CS
Agha	2019	278/305 (91.1%)	Yes						X	During labour and birth	Study excluded women with CS
Al-Mandeel	2013	49/182 (27%)	Study did not specify						X	Study did not specify	Study excluded women with CS
Balde	2020	Ghana: 697/836 (83.4%)	Study did not specify	X		X	X	X		Study did not specify	Study excluded women with CS
Balde	2020	Guinea: 608/644 (94.4%)	Study did not specify	X		X	X	X		Study did not specify	Study excluded women with CS
Balde	2020	Myanmar: 628/631 (99.5%)	Study did not specify	X		X	X	X		Study did not specify	Study excluded women with CS
Balde	2020	Nigeria: 561/543 (96.8%)	Study did not specify	X		X	X	X		Study did not specify	Study excluded women with CS
Baldissero tto	2016	Combined yes and partial: 3200/4162 (6.9%)	Yes						X	During labour and birth	Study excluded women with CS
BarrosGui da	2017	455/520 (87.5%)	Study did not specify						X	During labour only	Study excluded women with CS

Authors	Year	Coverage of labour companionship	Woman allowed companion of choice	Type of companion present during labour and/or birth						Timing of companionship allowed	Companion allowed during CS
				Any family member or friend (gender not specified)	Family member or friend (female only)	Husband or partner only	Doula	Traditional birth attendant	Study did not specify		
Berhane	2019	155/398 (38.9%)	Study did not specify						X	Study did not specify	Study excluded women with CS
BeyeneGetahun	2020	56/407 (13.7%)	Study did not specify						X	During labour and birth	Study excluded women with CS
Bezerra	2019	138/768 (17.9%)	Study did not specify						X	During labour, birth and postnatal	Study excluded women with CS
Bharti	2021	0% (baseline data)	No						X	Companion not allowed	Study excluded women with CS
Blanc	2016	644/662 (97.3%)	Study did not specify	X						During labour, birth and CS	Yes
Cederfeldt	2016	3/164 (1.8%)	Study did not specify						X	During labour and birth	Study excluded women with CS
Chaote	2021	59%	Study did not specify						X	Study did not specify	Study excluded women with CS
Cheung	2011	0/226 (0%)	Yes	X		X				During labour only	Study excluded women with CS
Cortes	2018	128/140 (91.4%)	Yes						X	Study did not specify	Study excluded women with CS
deMouraAlves	2019	19/459 (4.1%)	Study did not specify						X	During birth only	Study excluded women with CS

Authors	Year	Coverage of labour companionship	Woman allowed companion of choice	Type of companion present during labour and/or birth						Timing of companionship allowed	Companion allowed during CS
				Any family member or friend (gender not specified)	Family member or friend (female only)	Husband or partner only	Doula	Traditional birth attendant	Study did not specify		
Dim	2011	0/395 (0%)	No						X	Companion not allowed	Study excluded women with CS
Donati	2021	51.9%	Study did not specify						X	During birth and CS	Yes
Drysdale	2021	3/212 (1.42%)	Yes			X				During birth only	Study excluded women with CS
Galle	2019	1/520 (0.2%)	Yes			X				During labour and birth	Study excluded women with CS
Gutiérrez-Martín	2020	349/368 (94.8%)	Study did not specify						X	Study did not specify	Study did not specify
Hajizadeh	2020	9/334 (2.7%)	Study did not specify				X			Study did not specify	Study excluded women with CS
He	2012	22131/82745 (26.7%)	Study did not specify				X			During birth only	Study excluded women with CS
Hoogenboom	2015	20/20 (100%)	Study did not specify		X	X		X		During labour and birth	Study excluded women with CS
HunieAsratie	2021	14.6%	Yes		X	X				During labour only	No
Kalisa	2016	78/350 (22.3%)	Study did not specify			X				During labour only	Study excluded women with CS

Authors	Year	Coverage of labour companionship	Woman allowed companion of choice	Type of companion present during labour and/or birth						Timing of companionship allowed	Companion allowed during CS
				Any family member or friend (gender not specified)	Family member or friend (female only)	Husband or partner only	Doula	Traditional birth attendant	Study did not specify		
Kc	2020	10321/53872 (19.2%)	Study did not specify	X						During labour only	Study excluded women with CS
Kerebih	2020	8/378 (2.1%)	Yes						X	Study did not specify	Study excluded women with CS
Khalife-Ghaderi	2021	128/225 (56.9%)	Study did not specify						X	During labour only	Study excluded women with CS
Kiti	2021	591/865 (68%)	Yes	X	X	X				During labour only	Study excluded women with CS
Kozhiman nil	2014	5.9%	Study did not specify				X			During labour only	Study excluded women with CS
Liu	2021	54.2%	Study did not specify						X	During labour and birth	Study excluded women with CS
Liu	2021	3677/4192 (87.7%)	Study did not specify	X			X			Study did not specify	Study excluded women with CS
Lobo	2010	92.2%	Yes	X		X				During labour only	Study excluded women with CS
LohandosS antos	2017	718/827 (86.8%)	Study did not specify						X	During labour and birth	Study excluded women with CS
Maldie	2021	174/369 (47.2%)	Yes	X						During labour, birth and CS	Yes

Authors	Year	Coverage of labour companionship	Woman allowed companion of choice	Type of companion present during labour and/or birth						Timing of companionship allowed	Companion allowed during CS
				Any family member or friend (gender not specified)	Family member or friend (female only)	Husband or partner only	Doula	Traditional birth attendant	Study did not specify		
Manu	2021	Bangladesh: 374/387 (96.6%)	Study did not specify						X	During birth only	Study excluded women with CS
Manu	2021	Ghana: 17/134 (12.6%)	Study did not specify						X	During birth only	Study excluded women with CS
Manu	2021	Tanzania: 5/120 (4.1%)	Study did not specify						X	During birth only	Study excluded women with CS
MartinsFrancoMotta	2016	26/51 (51.0%)	Study did not specify						X	During labour and birth	Study excluded women with CS
Mocumbi	2019	2095/3778 (55.5%)	Study did not specify	X	X	X				Study did not specify	Study excluded women with CS
Mollard	2022	726/747 (97.2%)	Study did not specify			X				During birth and CS	Yes
Mollard	2021	862/885 (97.4%)	Study did not specify			X				During birth only	Study excluded women with CS
Mukamurigo	2019	1/435 (0.2%)	Study did not specify			X				During labour and birth	Study did not specify
Pereira dos Santos Moura	2016	54/160 (33.7%)	Yes	X	X	X				During labour, birth and postnatal	Study excluded women with CS
Perkins	2019	55.5%	Yes		X	X				During labour and birth	Study excluded women with CS

Authors	Year	Coverage of labour companionship	Woman allowed companion of choice	Type of companion present during labour and/or birth						Timing of companionship allowed	Companion allowed during CS
				Any family member or friend (gender not specified)	Family member or friend (female only)	Husband or partner only	Doula	Traditional birth attendant	Study did not specify		
Pervin	2018	99%	Study did not specify	X						Study did not specify	Study excluded women with CS
Rishard	2021	9.5%	Study did not specify						X	During birth only	Study excluded women with CS
RodrÃ-guezColl	2021	191/194 (98.4%)	Yes						X	During labour and birth	Study excluded women with CS
Santos	2019	7204/9135 (78.9%)	Study did not specify						X	Study did not specify	Study did not specify
Sauls	2010	161/185 (87.0%)	Study did not specify						X	During labour only	No
Sheferaw	2017	195/240 (81.2%)	Study did not specify						X	During labour and birth	Study excluded women with CS
Spaich	2013	320/335 (95.5%)	Study did not specify	X		X				Study did not specify	Yes
Stanton	2013	31.4%	Study did not specify						X	During labour and birth	Study did not specify
Tempfer-Bentz	2011	1608/2247 (71.6%)	Yes		X	X				During labour, birth and CS	Yes
Tesfaye	2016	281/430 (65.3%)	Study did not specify	X						During birth only	Study excluded women with CS
Thapa	2013	78.2%	Study did not specify			X				During birth only	Study excluded women with CS

Authors	Year	Coverage of labour companionship			Woman allowed companion of choice	Type of companion present during labour and/or birth						Timing of companionship allowed	Companion allowed during CS
						Any family member or friend (gender not specified)	Family member or friend (female only)	Husband or partner only	Doula	Traditional birth attendant	Study did not specify		
Tomasi	2021	3254/ 3573 (91.1%)			Study did not specify						X	During labour, birth and postnatal	Study did not specify
Vaz	2014	88%			Study did not specify			X				Study did not specify	Study excluded women with CS
Weeks	2017	1234/1651 (74.3%)			Yes						X	During labour only	No
		Labour	Birth	Caesarean									
Afulani	2018	588/751 (78.3%)	192/751 (25.6%)	-	Study did not specify	X	X	X				During labour, birth and postnatal	Study excluded women with CS
Diniz	2014	16,739/23,879 (70.1%)	10,053/23,879 (42.1%)	-	Yes		X	X	X			During labour, birth and postnatal	Study excluded women with CS
Dynes	2019	418/935 (44.7%)	112/935 (11.9%)	-	Study did not specify	X		X		X		During labour and birth	Study excluded women with CS
Dynes	2018	418/935 (44.7%)	112/935 (11.9%)	-	Study did not specify	X		X		X		During labour and birth	Study excluded women with CS
Lopes	2021	359/372 (96.5%)	354/372 (95.2%)	-	Study did not specify	X		X				During labour and birth	Study excluded women with CS
MachadodosAnjos	2019	568/586 (96.9%)	Birth/CS: 561/586 (95.7%)	-	Yes	X	X	X				During labour, birth and postnatal	Study excluded women with CS

Authors	Year	Coverage of labour companionship			Woman allowed companion of choice	Type of companion present during labour and/or birth						Timing of companionship allowed	Companion allowed during CS
						Any family member or friend (gender not specified)	Family member or friend (female only)	Husband or partner only	Doula	Traditional birth attendant	Study did not specify		
Monguilhott	2018	1021/1975 (51.7%)	693/1759 (39.4%)	108/310 (34.8%)	Study did not specify						X	During labour, birth and CS	Yes
Oluoch-Aridi	2021	67/303 (22.1%)	49/302 (16.2%)	-	Yes						X	During labour and birth	Study excluded women with CS
Leal	2019	6452/13469 (47.9%)	Vaginal birth: 3801/11509 (33.0%)	-	Study did not specify						X	During labour only	Study excluded women with CS
Binfa	2016	69.60%	Third stage: 86.0%	-	Study did not specify						X	During labour, birth and CS	Yes
		**Presence of companion**	**Doula support**										
daMattaMachadoFernandes	2021	452/535 (84.5%)	145/541 (26.8%)		Yes	X			X			During labour, birth and CS	Yes
dosSantos Moura	2020	302/335 (90.1%)	71/335 (21.2%)		Study did not specify						X	Study did not specify	Study excluded women with CS
Giordano	2019	580/580 (100%)	438/580 (75.5%)		Yes	X			X			During birth only	Study excluded women with CS
		**Husband/ partner**	**Doula support**	**Another family/**									

Authors	Year	Coverage of labour companionship			Woman allowed companion of choice	Type of companion present during labour and/or birth						Timing of companionship allowed	Companion allowed during CS
						Any family member or friend (gender not specified)	Family member or friend (female only)	Husband or partner only	Doula	Traditional birth attendant	Study did not specify		
				friend/ companion									
Declercq	2014	77%	6%	46%	Study did not specify	X		X	X			During labour only	Study excluded women with CS
Menhart	2017	226/301 (75.1%)	2/301 (0.7%)	Other companion: 37/301 (12.3%)	Study did not specify	X		X	X			Study did not specify	Study excluded women with CS
Simon	2016	86.60%	3.8%	2.6%	Study did not specify	X		X	X			During labour, birth and CS	Yes
		Pre-delivery room	Delivery/ CS room										
Goncalves Ade	2015	97.10%	90.60%		Study did not specify	X		X	X			During labour, birth, CS, and postpartum	Yes
Singh	2021	378/823 (45.9%)	164/527 (31.1%)		Study did not specify						X	During labour, birth and postnatal	Study excluded women with CS
